# Higher Risk of Mortality in Intentional Traumatic Injuries; A Multivariate Regression Analysis of a Trauma Registry

**DOI:** 10.30476/BEAT.2020.46450

**Published:** 2020-04

**Authors:** Sait Saif, Yahya Ibrahim, Peyman Bakhshayesh

**Affiliations:** 1 *Imperial College Health Care, St Mary´s Hospital, London UK*; 2 *Karolinska Institute, Department of Molecular Medicine and Surgery, Imperial College Health Care, St Mary´s Hospital, London UK*

**Keywords:** Injury, Intentional, Trauma, Mortality, Survival

## Abstract

**Objectives:**

To assess whether intentional traumatic injuries are associated with higher mortality rate when compared to unintentional injuries.

**Methods:**

Data from SweTrau (Swedish National Trauma Registry). Information regarding age, gender, injury severity score (ISS), new injury severity score (NISS), Glasgow coma scale (GCS), systolic blood pressure, and respiratory rate were collected via “SweTrau”. “Mortality within 30 days of injury” was defined as having been registered as dead within 30 days following the injury. Intentional injuries compared to non-intentional injuries. Multivariate regression analysis was conducted. Stepwise forward and backward regression was conducted.

**Results:**

A total number of 3875 patients were included. There were 3613 (93%) non-intentional and 262 (7%) intentional patients. The 30-day mortality rate was higher in the intentional group compared to non-intentional group, 10% *vs.* 4% (*p*<0.001). Patients in the intentional group were younger than the non-intentional group, at 39±18 vs. 47±21 years old (*p*<0.001). In both, the forward and backward tests injury intention remained statistically significant with OR 2 (CI 1.1-3.7). Shock (OR 4.7, CI 2.9-7.8), Severe Head Injury (OR 8.9, CI 5.3-14.7), Age ≥ 60 (OR 6.7, CI 4.1-10.8), ISS ≥16 (OR 10.8, CI 6.9-16.9) and ASA (OR 3.5, CI 2.2-5.7) were other factors affecting mortality.

**Conclusion:**

Injury intention was an independent factor contributing to mortality in our study. This particular cohort needs further attention during trauma management with a holistic insight to improve their survival.

## Introduction

Despite significant advances in medicine, mortality in trauma patients still remains high, and contributes to 4-12% of total mortality in Europe or the United States [1, 2]. Between 2000-2010 deaths from trauma increased by 22.8% in the United States, whereas there was decrease in deaths from cancer and heart disease during the same time period [3].

There are several predictive models to identify risk factors for mortality in the context of trauma, such as Trauma Injury Severity Score (TRISS), Revised Trauma Score (RTS), and Injury Severity Score (ISS) [4]. Most of these models identify physiological parameters such as low GCS, presence of shock and on-going hemorrhage, or organ related injuries as potential risk factors for mortality [4-8]. Intentional injuries (i.e. self-inflicted or deliberate assault) make up 25% of all injuries worldwide and present as high energy polytrauma patients [1, 9]. Additionally they often have underlying mental health issues [10-12]. Although all of these factors are in themselves risk factors, intentional injury itself has not been investigated as a risk factor contributing to mortality previously. We aimed to assess whether intentional injuries are a cause of death while taking other traditional factors as ISS, GCS, Shock and Age into consideration.

## Material and Methods

*Study population*

Following formal approval from SweTrau, data was collected from all trauma patients admitted to Karolinska University Hospital, Trauma Centre via “SweTrau” which is Swedish National Trauma Registry. Study period was from 2013-2015, with 1-year extension to report mortality. Patients with intentional injuries were identified and compared with trauma patients with non-intentional injuries as a control group.

*Study protocol*

Information regarding age, gender, ISS, new injury severity score (NISS), GCS, systolic blood pressure, respiratory rate etc. were collected via “SweTrau”. “Mortality within 30 days of injury” was defined as having been registered as dead within 30 days following the injury. Data from “SweTrau” and The Swedish Population Registry were cross-referenced in order to collect data regarding the date of fatal outcome for each patient. “High energy” was defined as an ISS≥16. “High age” in trauma was defined as age≥60. “Shock” was defined as systolic blood pressure ≤90mmHg. “Severe head injury” was defined as GCS≤8.

*Ethics*

Ethical approval was achieved via Stockholm´s ethical committee, (Dnr. 2016/383-31/4) to investigate traumatic pelvic ring injuries initially. The ethical committee approved the data extraction for all traumas. Results of current study are extracted from the same material. SweTrau directory have formally approved the material of the current study. New ethical application was not necessary.

*Statistics*

Student T-test was used for parametric variables with normal distribution. Shapiro-Wilk test was used to test normal distribution. Non-parametric variables were analyzed using Mann-Whitney U-test. Categorical variables were analyzed using Chi-square test. Traditional factors affecting mortality with different distribution between the two groups were analyzed using univariate regression analysis. Forward and backward multivariate regression analysis with Wald correction was conducted. The model was tested using Hosmer-Lemeshow goodness of fit. Nagelkerke R-square was used was used describe impact of final model on outcome. A p-value under 0.05 was considered as statistically significant. All tests were two sided.

## Results

A total number of 3875 patients were included. There were 3613 (93%) non-intentional and 262 (7%) intentional patients. The 30-day mortality rate was higher in the intentional group compared to non-intentional group, 10% vs. 4% (*p*<0.001). Patients in the intentional group were younger than the non-intentional group, at 39±18 vs. 47±21 years old (*p*<0.001). There were more men involved in Intentional Injury group 69% vs 65%, however this was not statistically significant (*p*=0.142).

Patients with intentional injury were more severely injured, with a median ISS of 10 vs. 5 (*p*<0.001). Patients with intentional injury had lower GCS (*p*<0.001). Patients with intentional injury had lower systolic blood pressure in emergency department (ED), 130 vs. 140 (*p*<0.001). Patients with intentional injury had higher respiratory rate 19 vs. 18 (*p*<0.001). Intentional injuries were most commonly due to a fall from height (55%), whilst for non-intentional injuries, motor vehicle accidents were the most common mechanism of injury (51%) (*p*<0.001). Patients with intentional injuries were more likely to arrive in shock in the emergency department, 20% vs. 6% (*p*<0.001), (Table 1). In univariate regression analysis, patients with intentional injuries had a 30-day mortality risk three times higher as compared with trauma patients with non-intentional injuries (CI, 1.9-4.5) (Table 2). Comparison of traditional factors affecting mortality in trauma patients showed that patients in shock had an OR of 5.8 (CI 3.9-8.6), ISS≥16 OR 15.6 (CI 10.5-13.1), GCS < 9 OR 13.4 (CI 9.1-19.6), Age ≥ 60 OR 8.3 (CI 5.9-11-6), ASA 3-4 vs. ASA 1-2 OR 7.8 (CI 5.6-10.8) (Table 2).

A multivariate regression analysis in a forward and backward stepwise fashion with Wald correction was conducted. In both, the forward and backward tests injury intention remained statistically significant with OR 2 (CI 1.1-3.7). The final model showed that Shock (OR 4.7, CI 2.9-7.8), Severe Head Injury (OR 8.9, CI 5.3-14.7), Age ≥ 60 (OR 6.7, CI 4.1-10.8), ISS ≥16 (OR 10.8, CI 6.9-16.9) and ASA (OR 3.5, CI 2.2-5.7) were other factors affecting mortality. The final model was checked regarding goodness of fit with Hosmer-Lemeshow test showing a *p*-value of 0.147 and was accepted. Nagelkerke R square showed that 44% mortality could be described by these six factors.

## Discussion

The main finding from this study was that Intentional injury was an independent risk factor of mortality. Overall the mortality rate in the intentional trauma group was higher (10%) than the non-intentional group (4%) (*p*<0.001). Regression analysis revealed that intentional injury was a predictor of mortality and that patients had a three times higher odds of dying within 30 days compared to trauma patients with non-intentional injuries. Patients with intentional injury were more severely injured, had higher ISS, lower GCS and were more likely to be in shock in the emergency department. This is certainly consistent with pre-existing models for predicting mortality, based on parameters such as ISS [4] . 

There are a number of reasons to explain this higher observed mortality rate for intentional trauma patients. Mechanism of injury may explain this, with 55% of intentional trauma patients having sustained a fall from height, whereas the majority of the unintentional injury patients were involved in motor vehicle accidents. A fall from height has reportedly higher mortality compared to motor vehicle accident [10]. Intentional injury is often considered a chronic disease as many of these patients have already sustained trauma from previous interpersonal violence or attempted self-harm [13]. This high re-admission rate raises several issues. Repeat injuries tend to occur quickly. Smith et al found that patients with intentional trauma averaged 8 months between injuries and that patients with fatal trauma averaged 18.8 months between injury and death [14]. Studies have shown that multiple socioeconomic risk factors are associated with trauma. These include male sex, unemployment, low income, drug and alcohol use, previous suicide attempts and mental health issues [15-17]. Long term studies exploring the relationship between morbidity and mortality following frequent re-admissions in intentional injuries may be illuminating [12] . 

Patients with mental health issues are often poorly compliant with treatment. It has been shown that mental health is a predictor for poor physical health and therefore as a victim of trauma might be less likely to survive [18, 19]. One of the ways of reducing mortality in trauma patients is through a holistic approach to identifying pre-existing mental health in these patients, and treating it through psychological programs [20].

In conclusion, this study has identified that intentional injury is a significant risk factor for mortality in trauma patients. Studies have shown that these groups of patients come from a vulnerable high risk background, have poorer health and mental health illness. These may all be contributing factors leading to increased 30-day mortality and this increased risk should be considered by clinician treating such patients. These patients need special consideration, and a holistic approach to management of their injuries to improve survival.

**Table 1:** General patient characteristics between intentional and non-intentional injuries

**Total (N %)** **3875 (100%)**	**Intentional Injury** **262 (7%)**	**Non-Intentional Injury** **3613 (93%)**	**p-value**
Age (Mean±SD)	39±18	47±21	<0.001
Female, number (%)	82 (31)	1256 (35)	0.142
ISS^a^ (Median, IQR)	10, 2-25	5, 1-13	<0.001
NISS^b^ (Median, IQR)	12, 3-34	6, 3-17	<0.001
GCS^c^ (Median, IQR)	15, 14-15	15, 15-15	<0.001
HLOS^d^ (Median, IQR)	2, 1-13	1, 0-5	<0.001
ICULOS^e^ (Median, IQR)	0 (0-1)	0 (0-0)	<0.001
ED SBP^f^ (Median, IQR)	130, 111-147	140, 126-158	<0.001
ED RR^g^ (Median, IQR)	19, 15-25	18, 15-20	<0.001
30-Day Mortality (N %)	26 (10)	132 (4)	<0.001
Injury Mechanism (N %)			<0.001
MVA^h^	20 (8)	1841 (51)	
High Fall	144 (55)	815 (23)	
Low Fall	1(0.2)	664 (18)	
Others	97 (37)	293 (8)	
Pre-Injury ASA^i^ 3-4 (N %)	20 (8)	468 (13)	0.015
Shock (N %)	51 (20)	209 (6)	<0.001
ISS^a^≥16 (N %)	107 (41)	744 (21)	<0.001
Age ≥ 60 (N %)	18 (7)	582 (16)	<0.001
GCS^c^ < 9 (N %)	18 (7)	152 (4)	0.042

^a^ISS: Injury Severity Scale Score; ^b^NISS: New Injury Severity Scale Score; ^c^GCS: Glasgow Coma Scale Score; ^d^HLOS: Hospital Length of Stay; ^e^ICULOS: ICU Length of Stay; ^f^ED SBP: Emergency Department Systolic Blood Pressure; ^g^ED RR: Emergency Department Respiratory Rate; ^h^MVA: Motor Vehicle Accident; ^i^ASA: American Society of Anesthesiologists

**Table 2:** Univariate analysis of factors affecting 30-Day mortality

	**Odds Ratio**	**Confidence Interval**
Intentional Injury	3	1.9-4.5
Shock	5.8	3.9-8.6
ISS^a^≥16	15.6	10.5-23.1
GCS^b^ < 9	13.4	9.1-19.6
Age ≥ 60	8.3	5.9-11.6
ASA^c^ 3-4	7.8	5.6-10.8

^a^ISS: Injury Severity Scale Score; ^b^GCS: Glasgow Coma Scale Score; ^c^ASA: American Society of Anesthesiologists

## Conflict of interests:

The authors declare no conflict of interests
